# Safety and effectiveness of mepolizumab therapy in remission induction therapy for eosinophilic granulomatosis with polyangiitis: a retrospective study

**DOI:** 10.1186/s13075-022-02845-3

**Published:** 2022-06-29

**Authors:** Masanobu Ueno, Ippei Miyagawa, Takafumi Aritomi, Koichi Kimura, Shigeru Iwata, Kentaro Hanami, Syunsuke Fukuyo, Satoshi Kubo, Yusuke Miyazaki, Shingo Nakayamada, Yoshiya Tanaka

**Affiliations:** The First Department of Internal Medicine, School of Medicine, University of Occupational and Environmental Health, Japan, 1-1 Iseigaoka, Kitakyushu, 807-8555 Japan

**Keywords:** Eosinophilic granulomatosis with polyangiitis, Corticosteroid, Mepolizumab, Induction therapy

## Abstract

**Objectives:**

To investigate the safety and effectiveness of mepolizumab (MPZ), an anti-interleukin-5 antibody, as remission induction therapy for severe eosinophilic granulomatosis with polyangiitis (EGPA).

**Methods:**

The clinical courses of patients with severe EGPA over 6 months were retrospectively investigated and compared between patients treated with high-dose corticosteroid (CS) plus MPZ therapy (MPZ group, *n* = 7) and those treated with high-dose CS plus intravenous cyclophosphamide (IVCY) pulse therapy (IVCY group, *n* = 13). The primary endpoints were the MPZ retention rate and the IVCY completion rate. The secondary endpoints were adverse events and changes in the Birmingham Vasculitis Activity Score (BVAS), Vascular Damage Index (VDI), eosinophil counts, and concomitant CS doses, and the extent and rates of these changes were compared between the MPZ and IVCY groups.

**Results:**

Regarding the primary endpoints, the MPZ retention rate was 100%, and the IVCY completion rate was 61.5%. Regarding the secondary endpoints, adverse events were detected in 2/7 patients (28.6%) in the MPZ group and 7/13 patients (53.8%) in the IVCY group. BVAS and eosinophil counts significantly decreased in both groups at and after month 1, but there was no significant difference in the magnitude of changes between the two groups. VDI scores did not significantly increase in either group, and the degree of changes did not significantly differ between the two groups. Although concomitant CS doses significantly decreased at and after month 1 in both groups, the rates of decrease in CS doses at and after month 3 were significantly higher in the MPZ group.

**Conclusions:**

This study suggested that the use of MPZ as remission induction therapy for severe EGPA might be safe and effective for controlling disease activity and reducing CS doses.

**Supplementary Information:**

The online version contains supplementary material available at 10.1186/s13075-022-02845-3.

## Background

Eosinophilic granulomatosis with polyangiitis (EGPA) is preceded by asthma or allergic rhinitis and causes elevated peripheral eosinophil counts along with various symptoms, such as fever, arthralgia, pulmonary infiltrates, pericarditis, renal disorder, peripheral neuropathy, gastrointestinal hemorrhage, purpura, and other vasculitis symptoms [1, 2]. Corticosteroids (CS) are the mainstay of treatment for remission induction and maintenance therapy. In patients with severe vasculitis symptoms at the initial onset or relapse, treatment with CS alone may be insufficient. Remission induction therapy includes the administration of a high-dose CS in combination with an immunosuppressant (cyclophosphamide) or a CD20 monoclonal antibody (rituximab) [3–5]. However, in elderly patients and patients with poor general conditions, the concomitant use of immunosuppressants is often difficult because of the risk of adverse effects, such as cytopenia, cardiotoxicity, and increased susceptibility to infections.

It has recently been reported that mepolizumab (MPZ), an anti-interleukin (IL)-5 antibody, prolongs the remission period and allows the reduction of CS doses during EGPA treatment [6]. In 2018, MPZ was approved for coverage by the national health insurance system in Japan for the treatment of EGPA resistant to currently available therapies. We have previously reported the safety and effectiveness of MPZ in maintenance therapy in relapsing and refractory EGPA in clinical settings [7]. MPZ is an effective agent for maintenance therapy; moreover, since MPZ has been used as remission induction therapy for steroid-resistant EGPA, it has started to attract increased attention [8–11]. In the present study, we assessed the safety and effectiveness of MPZ at a dose of 300 mg/month in combination with high-dose CS as remission induction therapy for EGPA in real-world clinical settings. We compared MPZ with intravenous cyclophosphamide (IVCY) as remission induction therapy.

## Methods

### Patients

Between 2015 and 2021, remission induction therapy with high-dose CS at a dose of ≥ 0.8 mg/kg was administered to 20 patients with severe EGPA who met the diagnostic criteria for EGPA issued by the Japanese Ministry of Health, Labour and Welfare and the classification criteria issued by the American College of Rheumatology. Severe EGPA was defined based on the presence of life-threatening symptoms or organ dysfunction such as lung lesions, glomerulonephritis, central nervous system disorders, multiple mononeuropathy, cardiac lesions, gastrointestinal lesions, and ischemia of the four limbs [5].

To minimize the differences in treatments other than MPZ and IVCY between the two groups, this study targeted patients who were treated during the 3 years before and after 2018, when MPZ was approved for coverage by the national health insurance system in Japan (2015 to 2021). We included 7 patients who initiated high-dose CS therapy plus MPZ in or after 2018 and were treated for ≥ 6 months (MPZ group) and 13 patients who initiated high-dose CS therapy plus IVCY between 2015 and 2021 and were treated for ≥ 6 months (IVCY group, including two patients who initiated IVCY therapy in or after 2018). Although high-dose CS therapy was administered for 1 week in both groups, patients were started on MPZ or IVCY therapy due to resistance to CS. Drug selection was made based on shared decision-making between attending physicians and patients. In the MPZ group, CS was administered first followed by monthly MPZ (300 mg/month). In the IVCY group, CS was administered first followed by IVCY (10 to 15 mg/kg every 2 weeks for 6 doses) and subsequent oral administration of immunosuppressants (azathioprine in principle) at and after month 3 (Additional file 1: Fig. S1). In both groups, according to the protocol for tapering concomitant CS doses, CS (in prednisolone equivalent doses) was tapered by 10 mg every 2 weeks to 30 mg/day, then by 5 mg every 2 weeks to 15 mg/day and by 2.5 mg every 2 weeks to 5 mg/day. Depending on clinical courses, attending physicians were allowed to discontinue CS tapering or to increase CS doses at their own discretion.

The Human Ethics Review Committee of our university reviewed and approved this study (No. H27-014). Also, we complied with the Declaration of Helsinki. All participants provided informed consent prior to inclusion in the study. Details that might disclose the identity of study subjects were omitted.

### Clinical measurement

In this study, we retrospectively assessed the safety and effectiveness of MPZ and IVCY as remission induction therapy over a 6-month period after the initiation of both drugs. This study excluded patients treated with rituximab because it is not covered for the treatment of EGPA by the national health insurance system in Japan. The primary endpoints were (1) the retention rate at month 3 after the initiation of MPZ and (2) the IVCY completion rate. IVCY completion was defined as receiving all six doses of cyclophosphamide administered at 10 to 15 mg/kg every 2 weeks. The secondary endpoints were adverse events and the effectiveness of remission induction therapy in both groups. Effectiveness was assessed via the Birmingham Vasculitis Activity Score (BVAS) and each item, the Vascular Damage Index (VDI) and each item [12], eosinophil counts, and concomitant CS doses in both groups. In addition, the amount of decrease in BVASs, the amount of increase in VDI scores at months 3 and 6, the amount of decrease in peripheral eosinophil counts, and the amount and rate of decrease in concomitant CS doses were compared between the MPZ and IVCY groups.

### Statistical analysis

The data are expressed as median (interquartile range) or number (%). Differences between the groups were compared using Fisher’s exact test or the Wilcoxon rank-sum test.

The Wilcoxon signed-rank test was used to detect statistically significant differences between each group’s baseline data and those measured at months 1, 3, and 6. Differences between the groups (MPZ group vs. IVCY group) were compared using the Wilcoxon rank-sum test.

All reported *P* values were two-sided and were not adjusted for multiple testing. The level of significance was set at *P* < 0.05. All analyses were conducted using JMP Pro version 15 (SAS Institute Inc., Cary, NC) and GraphPad Prism 9 (GraphPad Software, San Diego, CA).

## Results

### Patient background

The characteristics of the patients are shown in Table 1. The characteristics of each patient at the diagnosis of EGPA are shown in Supplementary Table S1. No statistically significant differences were observed in BVAS and their scored items, eosinophil counts, and inflammatory responses at baseline between the two groups.

**Table 1:** Baseline characteristic of MPZ group (*n* = 7) and IVCY group (*n* = 13)

	MPZ group (***n*** = 7)	IVCY group (***n*** = 13)	***P*** value^*^
First case/recurrence case, *n* (%)	6 (85.7)/1 (14.3)	12 (92.3)/1 (7.7)	1.0000
Male/female	3/4	4/9	0.6514
Age	74.0 (63.0, 83.0)	60.0 (56.0, 76.5)	0.2042
Disease duration (months)	0 (0, 1)	0 (0, 0)	0.5327
Concomitant CS dose (PSL mg/day)	50.0 (50.0, 70.0)	60.0 (40.0, 65.0)	0.9362
BVAS	17.0 (14.0, 24.0)	17.0 (13.5, 22.5)	0.9051
BVAS items			
General	6 (85.7)	11 (84.6)	1.0000
Cutaneous	5 (71.4)	9 (69.2)	1.0000
ENT	5 (71.4)	7 (53.8)	0.6424
Cardiomyopathy	1 (14.3)	2 (15.4)	1.0000
Chest	5 (71.4)	8 (61.5)	1.0000
Abdominal	1 (14.3)	1 (7.7)	1.0000
Renal	2 (28.6)	3 (23.1)	1.0000
Sensory neuropathy	5 (71.4)	12 (92.3)	0.2702
Motor neuropathy	2 (28.6)	6 (46.2)	0.6424
ANCA positive status, *n* (%)	2 (28.6)	2 (15.4)	0.5868
White blood cell count (/μL)	15,200 (12,500, 25,500)	16,600 (14,650, 21,550)	0.4511
Absolute eosinophil count (/μL)	5760 (2475, 15,478)	7434 (1881, 11,273)	0.7214
CRP (mg/dL)	3.72 (0.80, 10.1)	8.50 (1.20, 13.9)	0.4054
ESR (mm/h)	44.0 (33.0, 78.0)	52.0 (23.0, 79.0)	0.8740
IgE (IU/mL)	997 (253, 1971)	1112 (436.5, 3586.5)	0.5006

Data are shown by median [quartile] or *n* (%). *P* values were determined by Fisher’s exact test or the Wilcoxon rank-sum test

*MPZ* mepolizumab, *IVCY* intravenous cyclophosphamide, *CS* corticosteroid (prednisolone or equivalent), *BVAS* Birmingham Vasculitis Activity Score, *ENT* ear, nose, and throat

*P < 0.05: MPZ group (*n* = 7) vs. IVCY group (*n* = 13)

### Safety of MPZ and IVCY

Regarding the primary endpoints, the retention rate at month 3 after the initiation of MPZ was 100%, and the IVCY completion rate was 61.5% (8/13 patients). Table 2 shows the adverse events detected in all patients. Adverse events were detected in 2/7 patients (28.6%) in the MPZ group and 7/13 patients (53.8%) in the IVCY group. The adverse events detected in the MPZ group were infections (bacterial bronchitis and respiratory syncytial virus infection) that were mild and relieved by outpatient treatment in both patients. No patients discontinued MPZ. Among the adverse events detected in the IVCY group, infections (pyogenic arthritis and candidemia) in two patients, hepatic function disorder in two patients, and decreased cardiac function in one patient resulted in the discontinuation of IVCY. Since one patient who developed candidemia died after 1 month of treatment, the comparison of effectiveness at month 6 was performed between seven patients in the MPZ group and 12 patients in the IVCY group.

**Table 2:** Adverse events of the MPZ group and the IVCY group

Case no.	Group	Adverse events
1	MPZ	None
2	MPZ	2 M: bacterial bronchitis (improved)
3	MPZ	None
4	MPZ	None
5	MPZ	None
6	MPZ	6 M: RS virus infection (improved)
7	MPZ	None
8	IVCY	1 M: cytomegalovirus infection (hospitalization treatment, improved) 1.5 M: purulent arthritis (hospitalization treatment, discontinuation of IVCY)
9	IVCY	3 w: candidemia (death, discontinuation of IVCY)
10	IVCY	None
11	IVCY	None
12	IVCY	2 M: cytomegalovirus infection (hospitalization treatment, improved) 3 M: *Aspergillus* pneumonia, nocardia pneumonia (hospitalization treatment, improved)
13	IVCY	None
14	IVCY	1.5 M: cytomegalovirus infection (hospitalization treatment, improved) 2 M: liver dysfunction (discontinuation of IVCY)
15	IVCY	None
16	IVCY	1 M: cytomegalovirus infection (hospitalization treatment, improved) 1 M: liver dysfunction (discontinuation of IVCY)
17	IVCY	None
18	IVCY	None
19	IVCY	2 w: cardiac dysfunction (discontinuation of IVCY)
20	IVCY	4 M: bacterial bronchitis (improved)

*MPZ* mepolizumab, *IVCY* intravenous cyclophosphamide, *M* month, *w* week

### Comparison of effectiveness between MPZ and IVCY

**Fig. 1:**
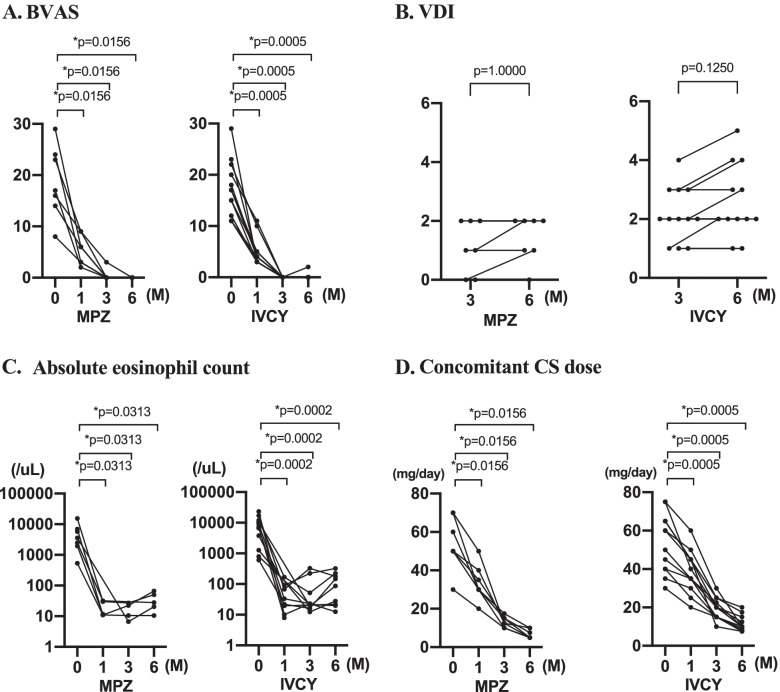
Changes in the effectiveness of remission induction therapy measured through four factors over 6 months. **A** BVAS. **B** VDI. **C** Peripheral eosinophil counts. **D** Concomitant CS doses. BVAS, Birmingham Vasculitis Activity Score; VDI, Vasculitis Damage Index; CS, corticosteroid; MPZ, mepolizumab; IVCY, intravenous cyclophosphamide. *P* values were determined by the Wilcoxon signed-rank test. *P < 0.05: baseline (month 0) vs. each observation points (months 1, 3, and 6)

In the MPZ group, the BVAS was 6.0 (3.0, 9.0) at month 1, 0 (0, 0) at month 3, and 0 (0, 0) at month 6, showing a significant reduction over time compared to baseline. In the IVCY group, the BVAS was 4.0 (3.0, 5.0) at month 1, 0 (0, 0) at month 3, and 0 (0, 0) at month 6, showing a significant decrease over time compared to baseline, as in the MPZ group (Fig. 1A).

**Fig. 2:**
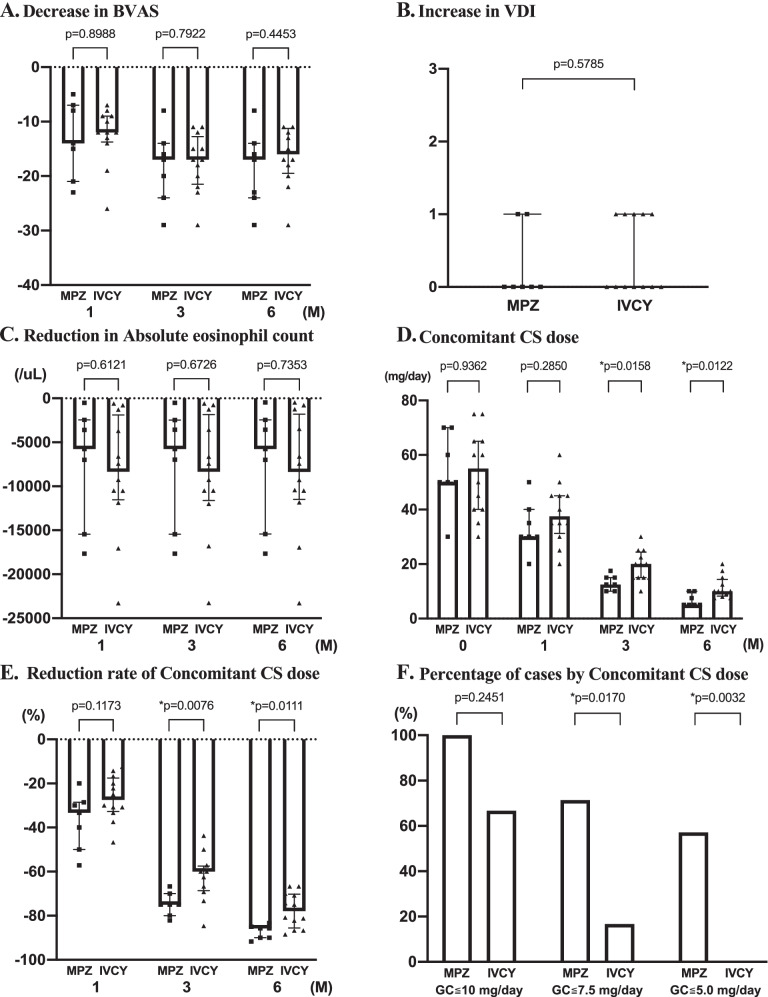
Comparison of the effectiveness of remission induction therapy over 6 months. **A** Decrease in BVAS. **B** Increase in VDI. **C** Reduction in peripheral eosinophil counts. **D** Concomitant CS dose. **E** Reduction rate of concomitant CS dose. **F** Percentage of cases by concomitant CS dose. BVAS, Birmingham Vasculitis Activity Score; VDI, Vasculitis Damage Index; CS, corticosteroid; MPZ, mepolizumab; IVCY, intravenous cyclophosphamide. *P* values were determined by the Wilcoxon rank-sum test. *P < 0.05: MPZ group vs. IVCY group

The amount of decrease in BVASs in the MPZ and IVCY groups was − 12.0 (− 13.8, − 9.0) and − 14.0 (− 21.0, − 7.0) at month 1, − 17.0 (− 24.0, − 14.0) and − 17.0 (− 21.5, − 12.8) at month 3, and − 17.0 (− 24.0, − 14.0) and − 17.0 (− 21.0, − 12.3) at month 6, respectively. The change in BVASs was not significantly different between the two groups at each observation point (Fig. 2A).

Table 3 shows the changes in organ dysfunctions on each BVAS item. At month 6, both groups exhibited improvement in general symptoms; skin manifestations; ear, nose, and throat manifestations; cardiac lesions; lung lesions; abdominal lesions; and renal lesions. Sensory or motor nerve involvement improved in some patients in both groups (sensory: MPZ 1/5 cases, IVCY 1/11 cases [*P* = 1.000]; motor: MPZ 1/2 cases, IVCY 1/6 cases [*P* = 0.4643]), but there were no differences between the groups. However, the disappearance of neurological symptoms was not observed in any patients within the study period.

**Table 3:** Changes in organ damage before and after the introduction of the MPZ group and the IVCY group

	MPZ group				IVCY group			
	0 M	1 M	3 M	6 M	0 M	1 M	3 M	6 M
General symptoms	6 (85.7%)	0	0	0	11 (91.7%)	0	0	0
Cutaneous manifestations	5 (71.4%)	1 (14.3%)	0	0	8 (66.7%)	4 (33.3%)	1 (8.3%)	0
ENT manifestations	5 (71.4%)	1 (14.3%)	1 (14.3%)	1 (14.3%)	7 (58.3%)	6 (50.0%)	2 (16.7%)	2 (16.7%)
Heart manifestations	1 (14.3%)	1 (14.3%)	0	0	2 (16.7%)	0	0	0
Chest manifestations	5 (71.4%)	3 (42.9%)	1 (14.3%)	0	7 (58.3%)	3 (25.0%)	1 (8.3%)	1 (8.3%)
Abdominal manifestations	1 (14.3%)	0	0	0	0	0	0	0
Renal manifestations	2 (28.6%)	0	0	0	2 (16.7%)	1 (8.3%)	0	0
Sensor nervous system manifestations	5 (71.4%)	5 (71.4%)	5 (71.4%)	5 (71.4%)	11 (91.7%)	11 (91.7%)	11 (91.7%)	11 (91.7%)
Motor nervous system manifestations	2 (28.6%)	2 (28.6%)	2 (28.6%)	2 (28.6%)	6 (46.2%)	6 (46.2%)	6 (46.2%)	6 (46.2%)

*ENT* ear, nose, throat; *MPZ* mepolizumab; *IVCY* intravenous cyclophosphamide

VDI scores were 1.0 (0, 2.0) at month 3 and 2.0 (1.0, 2.0) at month 6 in the MPZ group and 2.0 (1.3, 2.8) and 2.0 (2.0, 3.0), respectively, in the IVCY group. No significant differences between VDI scores at months 3 and 6 were observed in either group (Fig. 1B). The amount of increase in VDI scores from month 3 to month 6 was 0 (0, 1.0) in both MPZ and IVCY groups, showing no significant difference between the two groups (Fig. 2B). Table 4 shows the scored VDI items at months 3 and 6 in each patient.

**Table 4:** VDI items of the MPZ group and the IVCY group

Case no.	group	3 M	6 M
1	MPZ	Peripheral neuropathy, steroid-induced diabetes	Peripheral neuropathy, steroid-induced diabetes
2	MPZ	Compression fracture	Compression fracture, chronic bronchitis
3	MPZ		
4	MPZ	Steroid-induced diabetes	Steroid-induced diabetes, dyslipidemia
5	MPZ	Chronic cardiac failure, peripheral neuropathy	Cardiomyopathy, peripheral neuropathy
6	MPZ	Peripheral neuropathy, dyslipidemia	Peripheral neuropathy, dyslipidemia
7	MPZ		dyslipidemia
8	IVCY	Steroid-induced diabetes, chronic renal failure, hypertension, peripheral neuropathy	Steroid-induced diabetes, chronic renal failure, hypertension, stroke, chronic pulmonary aspergillosis
10	IVCY	Peripheral neuropathy	Peripheral neuropathy
11	IVCY	Hypertension, chronic renal failure	Hypertension, chronic renal failure
12	IVCY	Peripheral neuropathy	Peripheral neuropathy
13	IVCY	Steroid-induced diabetes, peripheral neuropathy, chronic bronchitis	Steroid-induced diabetes, peripheral neuropathy, chronic bronchitis, nocardia pneumonia
14	IVCY	Steroid-induced diabetes, peripheral neuropathy	Steroid-induced diabetes, peripheral neuropathy, chronic hepatitis
15	IVCY	Peripheral neuropathy	Peripheral neuropathy, dyslipidemia
16	IVCY	Steroid-induced diabetes, peripheral neuropathy	Steroid-induced diabetes, peripheral neuropathy
17	IVCY	Peripheral neuropathy, deep vein thrombosis	Peripheral neuropathy, deep vein thrombosis
18	IVCY	Vision impaired, peripheral neuropathy	Vision impaired, peripheral neuropathy
19	IVCY	Peripheral neuropathy, chronic cardiac failure	Peripheral neuropathy, chronic cardiac failure
20	IVCY	Chronic bronchitis, steroid-induced diabetes	Chronic bronchitis, steroid-induced diabetes

*MPZ* Mepolizumab, *IVCY* intravenous cyclophosphamide

Eosinophil counts were 11.0 (0, 30.0)/μL at month 1, 3.4 (0, 23.5)/μL at month 3, and 24.2 (0, 54.4)/μL at month 6 in the MPZ group, showing significant decreases from month 0 to month 1 and the subsequent points. In the IVCY group, eosinophil counts were 14.8 (0, 59.2)/μL at month 1, 20.4 (3.1, 44.9)/μL at month 3, and 25.1 (3.2, 170.9)/μL at month 6, also showing significant decreases at and after month 1 (Fig. 1C). The amount of decrease in eosinophil counts in the MPZ and IVCY groups was respectively − 5760 (− 15,448, − 2443)/μL and − 8340 (− 11,529, − 1879)/μL at month 1, − 5760 (− 15,451, − 2475)/μL and − 8349 (− 11,616, − 1837)/μL at month 3, and − 5760 (− 15,428, − 2447)/μL and − 8355 (− 11,489, − 1801)/μL at month 6, showing no significant differences between the two groups at each observation point (Fig. 2C).

The concomitant CS doses in the MPZ group were 30.0 (30.0, 40.0) mg/day at month 1, 12.5 (10.0, 15.0) mg/day at month 3, and 5.0 (5.0, 10.0) mg/day at month 6, showing significant decreases from month 0 to month 1 and the subsequent points. In the IVCY group, the concomitant CS doses were 37.5 (31.3, 45.0) mg/day at month 1, 20.0 (15.0, 24.4) mg/day at month 3, and 10.0 (8.3, 14.4) mg/day at month 6, also showing significant decreases at and after month 1 (Fig. 1D). When the concomitant CS doses at each observation point were compared between the two groups, the doses at and after month 3 were significantly lower in the MPZ group (Fig. 2D). The rates of decrease in CS doses in the MPZ and IVCY groups were respectively − 33.3% (− 50.0%, − 28.6%) and − 27.5% (− 32.7%, − 17.5%) at month 1, − 75.0% (− 80.0%, − 70.0%) and − 60.0% (− 68.6%, − 57.4%) at month 3, and − 85.7% (− 90.0%, − 85.0%) and − 77.9% (− 85.6%, − 85.0%) at month 6, showing significantly higher rates in the MPZ group at and after month 3 than in the IVCY group (Fig. 2E). The proportions of patients receiving CS doses of ≤ 10 mg/day, ≤ 7.5 mg/day, and ≤ 5.0 mg/day at month 6 were respectively 100%, 71.4%, and 57.1% in the MPZ group and 66.7%, 16.7%, and 0% in the IVCY group, showing significantly higher proportions of patients receiving CS doses of ≤ 7.5 mg/day and ≤ 5.0 mg/day in the MPZ group (Fig. 2F).

## Discussion

To the best of our knowledge, this is the first study comparing the safety and effectiveness of MPZ and IVCY as used in remission induction therapy with high-dose CS for highly active EGPA.

For the treatment of highly active EGPA, the concomitant use of cyclophosphamide or rituximab is recommended in addition to high-dose CS therapy [3–5]. However, it is difficult to administer potent immunosuppressive therapy, especially to elderly patients and patients with poor general conditions given the risk of infection. Therefore, these patients may not be able to receive sufficient remission induction therapy. MPZ, an IL-5 inhibitor, inhibits proliferation, differentiation, infiltration, activation, and survival of eosinophils [13, 14] but has minimal effect on lymphocytes and neutrophils. Despite the risk of exacerbating parasitic infection, MPZ appears to be associated with lower risks of bacterial and fungal infections. In real-world clinical settings, the incidence of infections related to MPZ used for the treatment of EGPA has been reported to be 0.9% [15]. In the present study, although some patients in the IVCY group discontinued treatment because of infections, the retention rate in the MPZ group was 100%. Regarding adverse events, no patients developed severe infections that required hospitalization. Thus, MPZ appeared to be a highly safe drug (Table 2).

Regarding effectiveness, BVASs and eosinophil counts started rapidly decreasing 1 month after treatment initiation in both groups, and no significant differences were observed between the two groups (Figs. 1 and 2). CSs are known to reduce human eosinophil counts through direct and indirect mechanisms and to be effective for controlling eosinophilic inflammation [16]. Thus, they might have contributed to rapid reductions in BVAS and eosinophil counts at and after 1 month of treatment. At month 6, BVAS and rates of decrease in BVAS did not significantly differ between the two groups, and changes in organ dysfunctions showed no marked differences. The MPZ therapy appeared to be as effective as the IVCY therapy.

CS doses were significantly lower, and rates of decrease in CS doses were significantly higher in the MPZ group than in the IVCY group at and after 3 months of treatment. EGPA is widely known to relapse during tapering of CS [17]. In the IVCY group, the completion rate was low, and the remission induction therapy administered was insufficient. This suggests that the lower rate of decrease in CS doses may have been due to concerns regarding the risk of relapse. Since CSs induce various complications including not only infections but also osteoporosis, diabetes mellitus, hypertension, dyslipidemia, and femur head necrosis, early dose reduction is preferable. Although no significant differences in VDI scores were observed between the two groups during the 6-month observation period in this study, CS doses were reduced to a significantly greater extent in the MPZ group during remission induction therapy. This may lead to a lower incidence of complications in the future. It is important, in the future, to determine whether CSs can be administered at low doses or discontinued without relapse for a long period of time and to observe whether VDI scores increase.

In this study, there was no significant difference in the improvement of neurological (sensory or motor) involvement between the groups. We had previously reported that the serum IL-5 levels of relapsing/refractory EGPA, even in the maintenance phase, were significantly higher than of healthy controls [7]. Namely, there is a possibility that sustained increase in IL-5 levels contributes to chronic organ involvement or treatment (CS) resistance. Therefore, extended observation may reveal a higher treatment effectiveness of MPZ even in neurological involvement within our cohort. Consistent treatment with MPZ, starting from an induction phase to maintenance phase, may be a novel treatment strategy enabling the prevention or avoidance of treatment resistance or chronic organ involvement, especially neurological involvement. In this study, there are several limitations to be noted. First, due to the small sample size, statistical power was insufficient, and our data was partly affected by randomization error. In addition, the long-term effects after remission induction therapy, including the effects during the maintenance phase, were not sufficiently assessed because this study focused on the short-term effects during remission induction therapy. That was a reason why the satisfied improvement of neurological disorders was not assessed within a short term. Further studies with larger sample size and longer observation periods would be warranted to assess the safety and effectiveness of MPZ after the remission induction phase.

## Conclusions

This study demonstrated that the use of MPZ in remission induction therapy for severe EGPA allowed safe control of disease activity and reduction of concomitant CS doses.

## Supplementary Information

**Additional file 1: Fig. S1.** Study design.

## Data Availability

Not applicable.

## Abbreviations

EGPA: Eosinophilic granulomatosis with polyangiitis
CS: Corticosteroids
MPZ: Mepolizumab
IL: Interleukin
IVCY: Intravenous cyclophosphamide
BVAS: Birmingham Vasculitis Activity Score
VDI: Vascular Damage Index
