# Supplementary figures and images for: Safety and effectiveness of mepolizumab therapy in remission induction therapy for eosinophilic granulomatosis with polyangiitis: a retrospective study
Source: Arthritis Res Ther. 2022 Jun 29;24:159. doi: 10.1186/s13075-022-02845-3 (PMC9241238; doi:10.1186/s13075-022-02845-3)

Supplementary Figure. 1

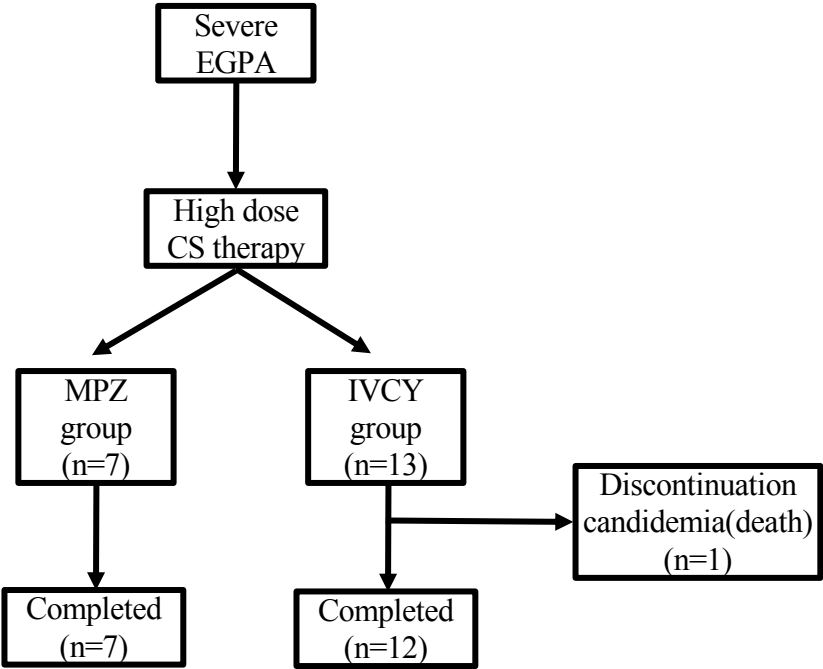

Supplement: Supplementary file 1 — Additional file 1: Fig. S1. Study design. [file 13075_2022_2845_MOESM1_ESM.pdf]
